# Performance controlled via surface oxygen-vacancy in Ti-based oxide catalyst during methyl oleate epoxidation

**DOI:** 10.1038/s41598-020-76094-2

**Published:** 2020-11-03

**Authors:** Supareak Praserthdam, Meena Rittiruam, Kanokpon Maungthong, Tinnakorn Saelee, Siriwimol Somdee, Piyasan Praserthdam

**Affiliations:** 1 High-Performance Computing Unit (CECC-HCU), Center of Excellence on Catalysis and Catalytic Reaction Engineering (CECC), Chulalongkorn University, Bangkok, 10330 Thailand; 2 Center of Excellence on Catalysis and Catalytic Reaction Engineering (CECC), Chulalongkorn University, Bangkok, 10330 Thailand

**Keywords:** Heterogeneous catalysis, Chemical engineering, Atomistic models, Chemical physics

## Abstract

The catalytic performance with high conversion and high selectivity of Ti-based oxide catalysts have been widely investigated. Besides, stability, which is an essential parameter in the industrial process, lacked fundamental understanding. In this work, we combined computational and experimental techniques to provide insight into the deactivation of P25 and TS-1 Ti-based oxide catalysts during the methyl oleate (MO) epoxidation. The considered deactivation mechanisms are fouling and surface oxygen vacancy (O_V_). The fouling causes temporary catalyst deactivation through active site blockage but can be removed via calcination in air at high temperature. However, in this work, the O_V_ formation plays an important role in the overall performance of the spent catalyst as the deactivated catalyst after regeneration, cannot be restored to the initial activity. Also, the effects of O_V_ in spent catalysts caused (i) the formation of more Ti^3+^ species on the surface as evident by XPS and Bader charge analysis, (ii) the activity modification of the active region on the catalyst surface as the reduction in energy gap (E_g_) occurred from the formation of the interstates observed in the density of states profiles of spent catalyst modeled by the O-vacant P25 and TS-1 models. This reduction in E_g_ affects directly the strength of Ti–OOH active site and MO bonding, in which high binding energy contributes to a low conversion because the MO needed an O atom from Ti–OOH site to form the methyl-9,10-epoxy stearate. Hence, the deactivation of the Ti-based oxide catalysts is caused not only by the insoluble by-products blocking the active region but also mainly from the O_V_. Note that the design of reactive and stable Ti-based oxide catalysts for MO epoxidation needed strategies to prevent O_V_ formation that permanently deactivated the active region. Thus, the interrelation and magnitude between fouling and O_V_ formation on catalyst deactivation will be investigated in future works.

## Introduction

Since chemical industries are shifting towards greener technologies, utilizing renewable feedstocks, e.g., biomass, overcomes the shortage of natural resources while reducing environmental is impacted by moving away from petroleum-based supply. In the plant-based oil-producing regions, especially in Southeast Asia, the palm oil is oversupplied. Thus, the conversion of palm oil to other higher-value products is of great interest^1^. One of the targeted products is green diesel, called the fatty acid methyl esters (FAME). It was produced either via the esterification reaction of animal or plant-based oil. ^2,3^ However, various energy policies in different countries that encourage the use of electric vehicles to lower the demand for biodiesel suppressed the demand for FAME. Hence, the epoxidation reaction can be the solution towards the utilization of the FAME producing higher-value chemicals, especially, epoxide products^4^ which are used in as plasticizers^5^, stabilizer in PVC, intermediates in polyurethane polyols ^6^, lubricants ^7^, cosmetics, precursors of various polymer ^8^, wood impregnation, biofuel additives ^9^, and in pharmaceuticals ^10^. So far, the low-temperature liquid-phase epoxidation reaction was described by Prileshajew, as shown in Supplementary Figures S1. The homogeneous reaction is catalyzed by percarboxylic acids which are formed in situ from the reaction between hydrogen peroxide and the carboxylic acid on soluble mineral acids such as H_3_PO_4_, HCl, or H_2_SO_4_^9,11^. In this work, an unsaturated FAME, methyl oleate (MO) is studied, where the targeted epoxide product is the methyl-9,10-epoxy stearate (ME). However, a homogeneous process that is used as catalysts in an industrial process causes corrosion from H_3_PO_4_, HCl, and H_2_SO_4_^12^, exhibiting low selectivity since the soluble mineral acid promotes side reactions, e.g., oxirane ring-opening ^1^. In addition, the catalyst reuse from a homogeneous process is impractical due to the difficult separation between the catalysts and products. Due to this, the Ti-based oxide catalysts are of interest for the epoxidation reaction. This commercial catalyst is available as titanium dioxide (TiO_2_) with a great number of the Ti–O–Ti moieties known as P25 ^1^. In addition, the ordered structure titanosilicate, TS-1 containing SiO_2_-doped TiO_2_ exhibiting high conversion and selectivity is considered a good candidate ^12^ because SiO_2_ help promote the dispersion of the TiO_2_^13^. The active center in FAME epoxidation reaction on the TiO_2_ is the titanium hydroperoxo species (Ti–OOH) formed via the reaction between the added hydrogen peroxide and the clean TiO_2_ surface. The Ti–OOH active sites converted MO to ME product and H_2_O as by-product illustrated in Figures S2 of the supplementary document. To achieve the highest performance for such catalysts, one must tackle its surface deactivation by understanding the underlying mechanism during the liquid-phase epoxidation reaction occurred at low temperature ^2^.

Therefore, we investigate the deactivation scheme on P25 and TS-1 Ti-based oxide catalysts during the liquid-phase MO epoxidation reaction at low temperature based on the evidence from experimental data, surface characterizations, and computational data obtained via the density functional theory-based (DFT) analysis ^14–19^. Ultimately, the information of such deactivation schemes would be used to construct the guideline towards the design of reactive and stable Ti-based oxide catalysts for epoxidation reactions.

## Results and discussion

**Figure 1:**
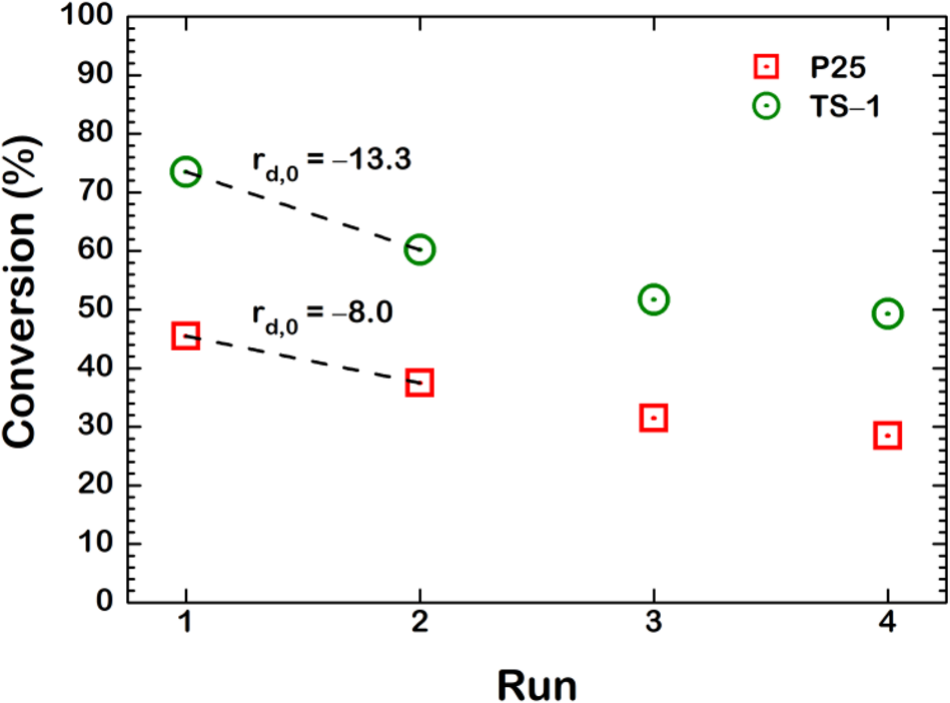
The methyl oleate conversion on P25 (red square) and TS-1 (green circle) catalysts during methyl oleate epoxidation reaction operated in a batch reactor.

To understand the deactivation of the P25 and TS-1 catalysts, we first determine their stability in terms of MO conversion during the epoxidation reaction on the fresh and used catalysts, as shown in Fig. 1. For the first batch, exactly, the TS-1 yielded higher conversion at 73.5% than that of the P25 at 45.5%. The deactivation is then pre-evaluated via the initial deactivation rate $(\mathrm{r}_{{\mathrm{d}},0})$ calculated from the change of MO conversion over the first and second batch, where the ${\mathrm{r}}_{{{\mathrm{d}},0}}$ is − 13.3 for the TS-1 and − 8.0 for the P25. Up to this point, the assumption towards the deactivation was proposed to be formed the fouling of either products or by-products, in which the carbon contents may occur in the used catalysts. This concern can be eliminated by the calcination method. As a result, the spent TS-1 and P25 catalysts from the 1st batch were calcined in air at 550 °C for 5 h to confirm the removal of the fouling species. This selected temperature is confirmed to avoid the phase change of our Ti-based oxide catalyst^20^. Thus, the regenerated catalysts should perform with comparable MO conversion to the fresh catalyst if the fouling is the main cause of deactivation.

The MO conversion of fresh catalysts was compared with that of with and without calcinated catalysts as shown in Table 1. It is suggested the removal of the fouling species via calcination can be observed from the increased MO conversion of calcined catalysts. In fact, we found that the MO conversion of the calcined ones was not regenerated back to the value of the fresh catalysts. This raised the concern about our assumption that fouling may not be only the main contributor to the deactivation mechanism. However, the catalyst surface transformation may play a role in the modification of active regions. We intended to investigate the changes in the number of surface species of the elements on the surface: Ti and O on both catalysts. Hence, the tracking of the surface transformation during the reaction was carried out via the XPS technique.

**Table 1:** The MO conversion of 1st run, 2nd without calcination, and 2nd run with calcination, for P25 and TS-1.

Catalyst	MO conversion (%)		
	1st run	2nd run without calcination	2nd run calcined at 550 °C
P25	45.5	37.5	42.4
TS*-*1	73.5	60.2	67.2

**Figure 2:**
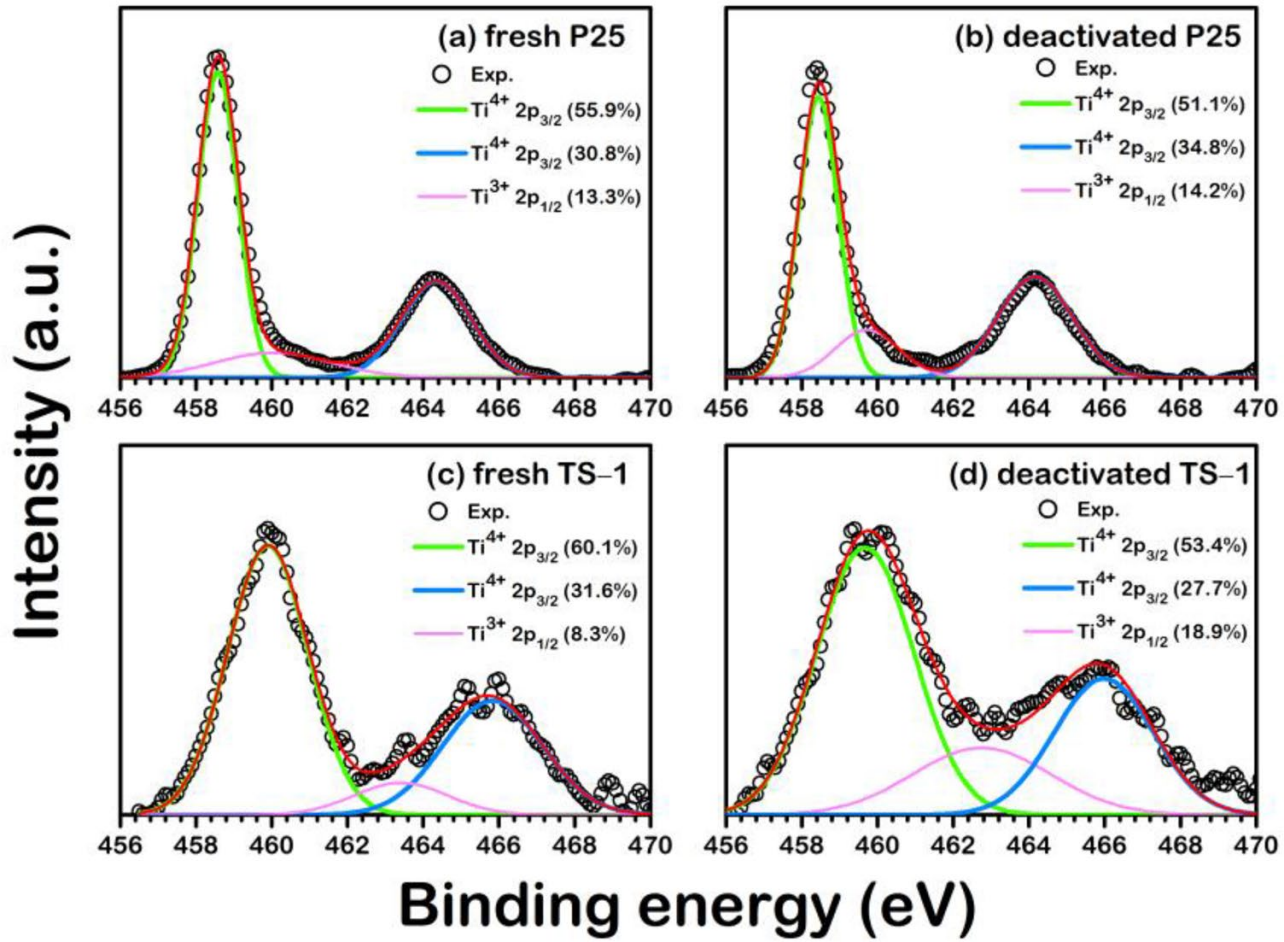
The high-resolution XPS spectra of Ti2p in (**a**) fresh P25, (**b**) deactivated P25, (**c**) fresh TS-1, and (**d**) deactivated TS-1.

**Figure 3:**
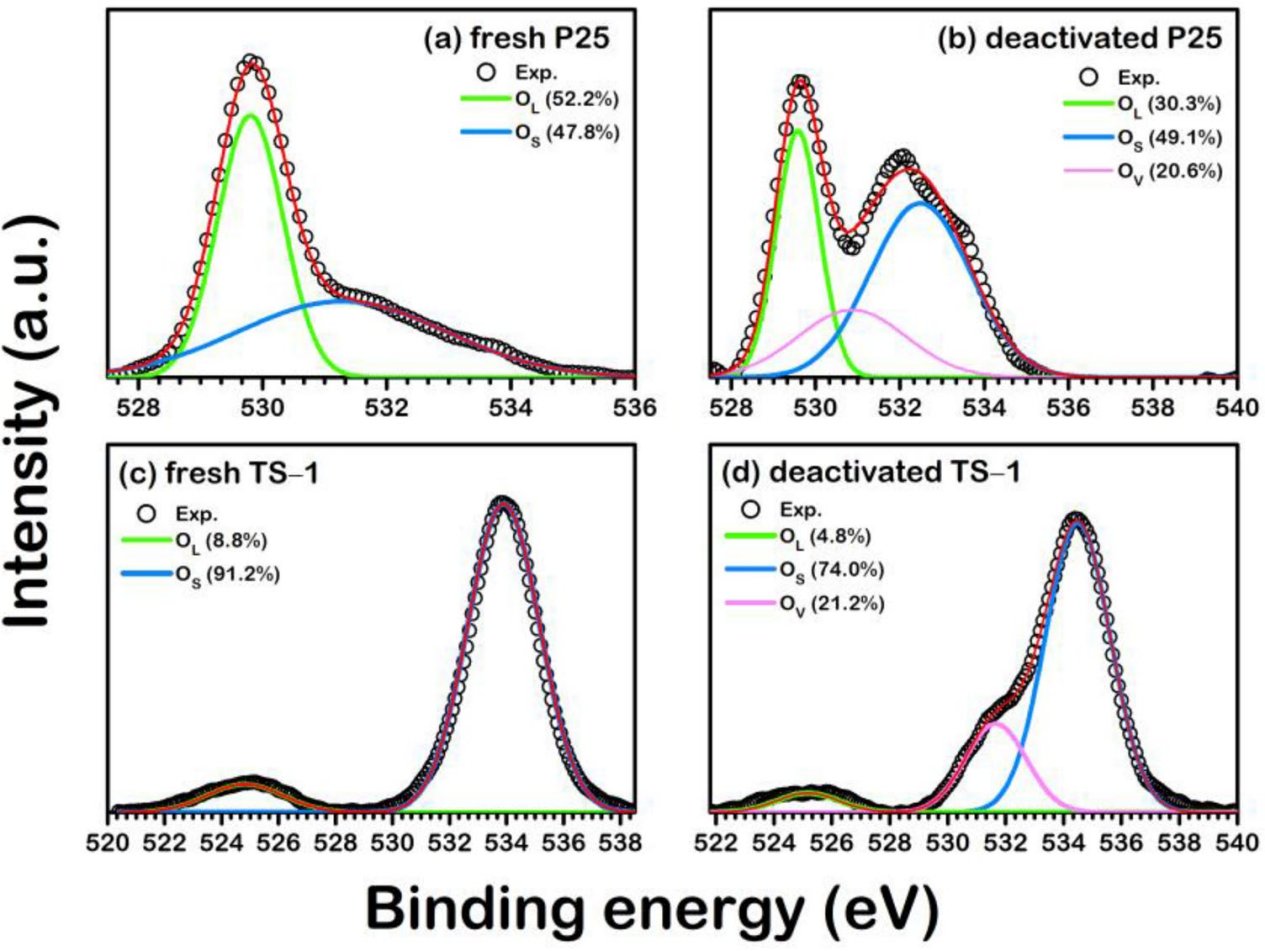
The high-resolution XPS spectra of O1s in (**a**) fresh P25, (**b**) deactivated P25, (**c**) fresh TS-1, and (**d**) deactivated TS-1.

The high-resolution XPS spectra profiles of P25 and TS-1 illustrated in Figs. 2 and 3 reveal the changes in the amount of Ti^3+^, Ti^4+^, and O vacancy on the catalyst surfaces. The analyzed XPS is implemented in the Supplementary document as Table S1. On the Ti species of the fresh catalysts, the Ti^4+^2p_3/2_ peaks were detected at 458.6 eV and 464.2 eV for P25, and 459.9 eV and 465.7 eV for TS-1. These peaks are in good agreement with the anatase TiO_2_^21^ and TS-1^22^. In addition, the Ti^3+^2p_1/2_ peak, which was classified to the Ti_2_O_3_ phase ^23^, occurred between the peaks of Ti^4+^2p_3/2_ at 460.4 eV for P25 and 463.4 eV for TS-1 catalysts. To track the evolution of the catalyst surface during MO epoxidation, the XPS profile in Fig. 2 was analyzed for the changes in the content of surface Ti^3+^ and Ti^4+^ species. From Fig. 2a, the fresh TS-1 possessed 8.3% of Ti^3+^ species, which is less than that of the P25 (13.3%), shown in Fig. 2c. On the profiles of spent catalysts shown in Fig. 2b,d, the Ti^3+^ species increased to 18.9% and 14.2% for TS-1 and P25, respectively. Besides, the changes in surface oxygen vacancies were investigated via the XPS, as shown in Fig. 3. The XPS profile indicates that there are three oxygen species: lattice oxygen (O_L_), sub oxide (O_S_), and oxygen vacancy (O_V_) that are also confirmed by Bharti et al.^23^ It was found in all fresh catalysts that only the O_L_ and O_S_ species were observed, while the O_V_ was found in all reused catalysts. The O_V_ observed in the deactivated ones is confirmed that the deactivation during MO epoxidation involves the removal of a surface oxygen atom. Therefore, we hypothesized the formation of these O_V_ modified the active region on the catalyst surface, resulting in the reduced activity which leads to reduce the MO conversion. To further clarify such argument on the deactivation mechanism, the DFT analysis was employed in the following section.

To investigate the role of O_V_ species formed on the surface of reused catalysts, we first modeled the P25 and TS-1 surface. From the XRD profile, shown in Supplementary Figures S3, the P25 exhibits anatase as the main phases, while rutile also hardly occurs. The TS-1 shows the ZSM-5-like the structure of high crystalline TS-1, in which the Ti peaks also appear in the TS-1 XRD profile. Therefore, the models in this study were designed based on the XRD profiles.

There are two different methods to design the P25 and TS-1 catalyst surfaces because of a different crystal structure. For the P25 surface, it is confirmed from the experiment that anatase TiO_2_ is the main phase. We first calculated the full geometry-optimization in the bulk anatase TiO_2_, in which the structural parameters are uncovered in Supplementary.

**Figure 4:**
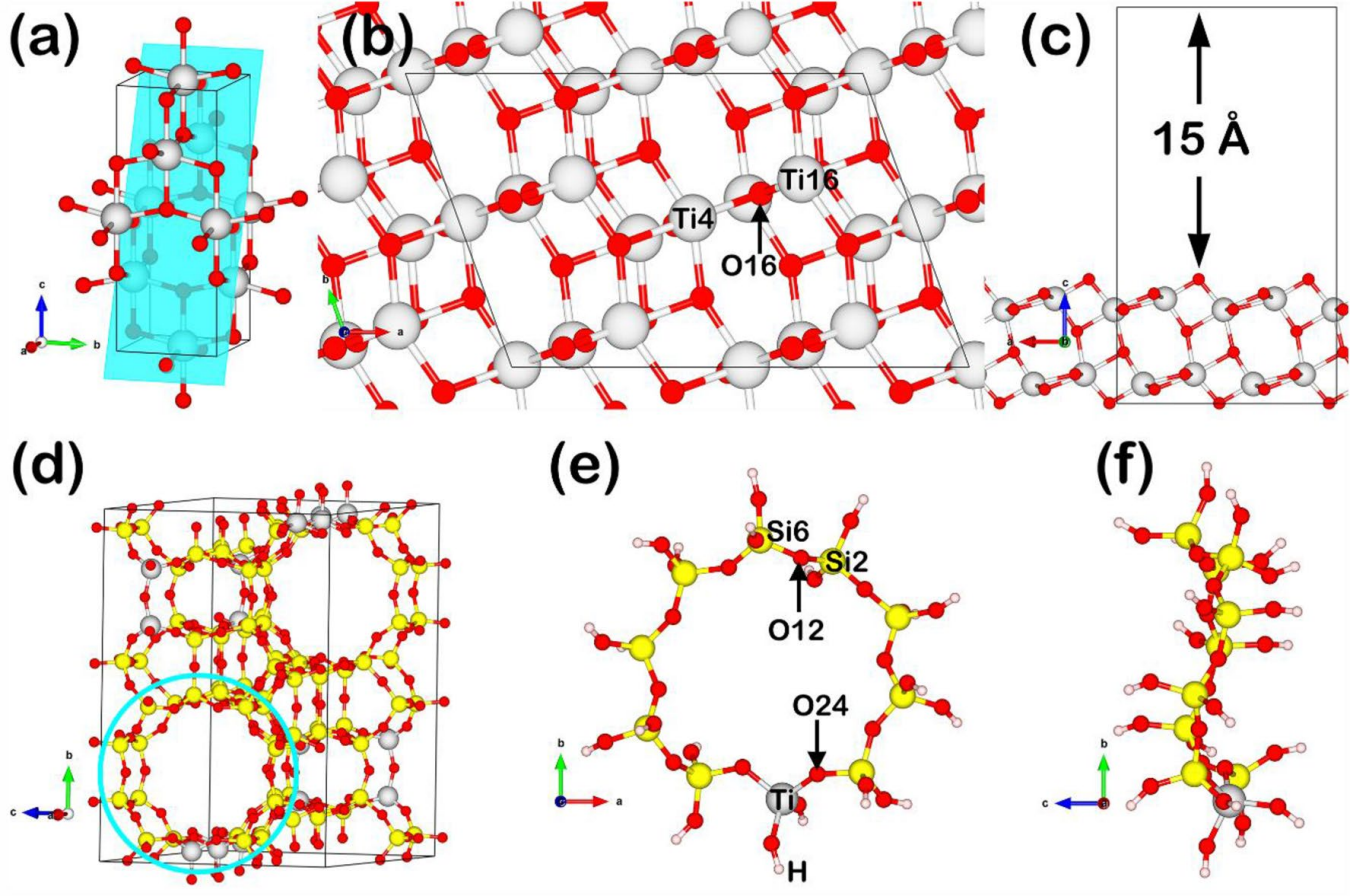
The P25 and TS-1 catalyst model. The P25 is represented by anatase TiO_2_, showing (**a**) bulk structure, and (**b**, **c**) top and side views of anatase TiO_2_ (101). The TS-1 is optimized through the TS-1 (ZSM-5, Ti-doped SiO_2_), including (**d**) bulk structure, and (**b**, **d**) front and side views of TS-1. The designed structure is described in the result and discussion section.

The optimized model of P25 is illustrated in Fig. 4a. The anatase TiO_2_ phase was represented by the TiO_2_(101) slab model referring to the model construction of Lazzeri et al. ^24^ who revealed such TiO_2_(101) surface to be the most stable surface of anatase. The surface model comprised of 48 atoms having the formula of Ti_16_O_32_ as 2 × 2 × 1 supercell (Fig. 4b), where the vacuum is set to 15 Å to avoid the interactions due to the periodicity (Fig. 4c). This surface model shows two Ti–O layers represented the unit cell of anatase TiO_2_ (101), where the increase of the Ti–O layer of this pattern not effects to density of states and still has the same energy gap. In the full geometry-optimization, we use the selective dynamics in order to allow the Ti–O top layer to free relax, while, the Ti–O bottom layer is fixed.

For the TS-1 model, it was designed by the Ti-doped ZSM-5-like structure based on the reported procedures in our previous works ^25,26^. In each 10 T ring, one Ti atom was substituted on one Si atom forming the TS-1 with Ti:Si ratio of 1:5 as illustrated in Fig. 4d. To represent well the real TS-1 structure, we performed the geometry optimization of the Si_80_Ti_16_O_192_ bulk structure shown in Fig. 4d. Because the reaction occurs in the 10 T ring rather than in other regions, the TS-1 surface model is also different from the P25. We thus scaled-down the TS-1 cluster represented by a 9.5-Å-diameter 10 T ring to be the reactive region participating in the reaction modeled in a 25 × 25 × 15 Å^3^ cubic system.

In addition, to represent the rest of the TS-1 framework, after the optimization of the bulk structure, one hydrogen atom was added to all terminal O atoms of the 10 T ring. All dangling OH groups were fixed to the lattice parameter of the bulk to represent the TS-1 structure before being further optimized. In the geometry optimization, we also used the selective dynamics method to allow atoms to relax except the OH groups fully. The optimized TS-1 surface model is shown in Fig. 4e,f.

On the representation of the deactivated surface caused by O_V_ formation, the O_V_ sites on the P25 and TS-1 surfaces were created and modeled by removing an O atom in each model at the following locations: (1) O16 in TS-1, (2) O24 in P25 representing the O_V_ near a Ti-site (O_V,Ti_), and (3) O12 in P25 representing the O_V_ near a Si-site (O_V,Si_), in which the atomic position label is shown in Fig. 4b for P25 and Fig. 4e for TS-1.

**Figure 5:**
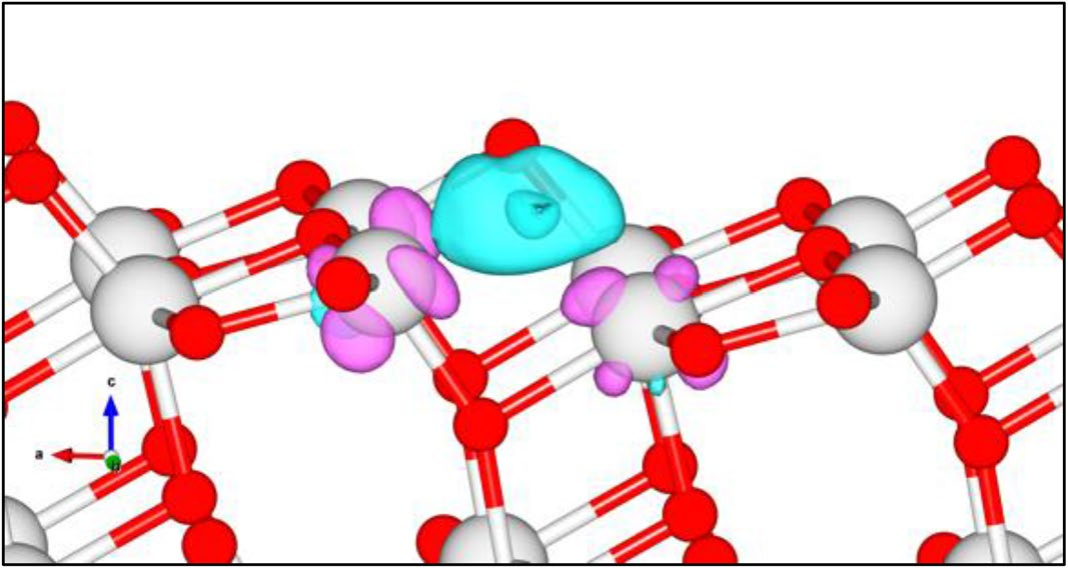
Electron transfer in terms of charge accumulation/depletion regions in the P25 surface with O vacancy. The blue regions represent charge accumulation, while the purple regions represent charge depletion.

Having all the modeled catalysts, the analysis of their electronic properties was carried out by the Bader charge analysis. On the P25 surface, it was found that the formation of O_V_ induced charge accumulation in the region between the two Ti atoms (Ti4 and Ti16) previously connected to the removed O atom, while the charge of both Ti atoms depleted as shown in Fig. 5. In addition, from the charge transfers of Ti4 and Ti16, labeled in the Fig. 4b, decreased from + 2.41 |*e*| and + 2.36 |*e*| to + 2.03 |*e*| and + 1.99 |*e*|, respectively. This decrease in charge transfer correlated with the decrease in an oxidation state of Ti from Ti^4+^ to Ti^3+^ verified by XPS. Hence, the model of P25 can represent well the P25 used in the experiment.

**Figure 6:**
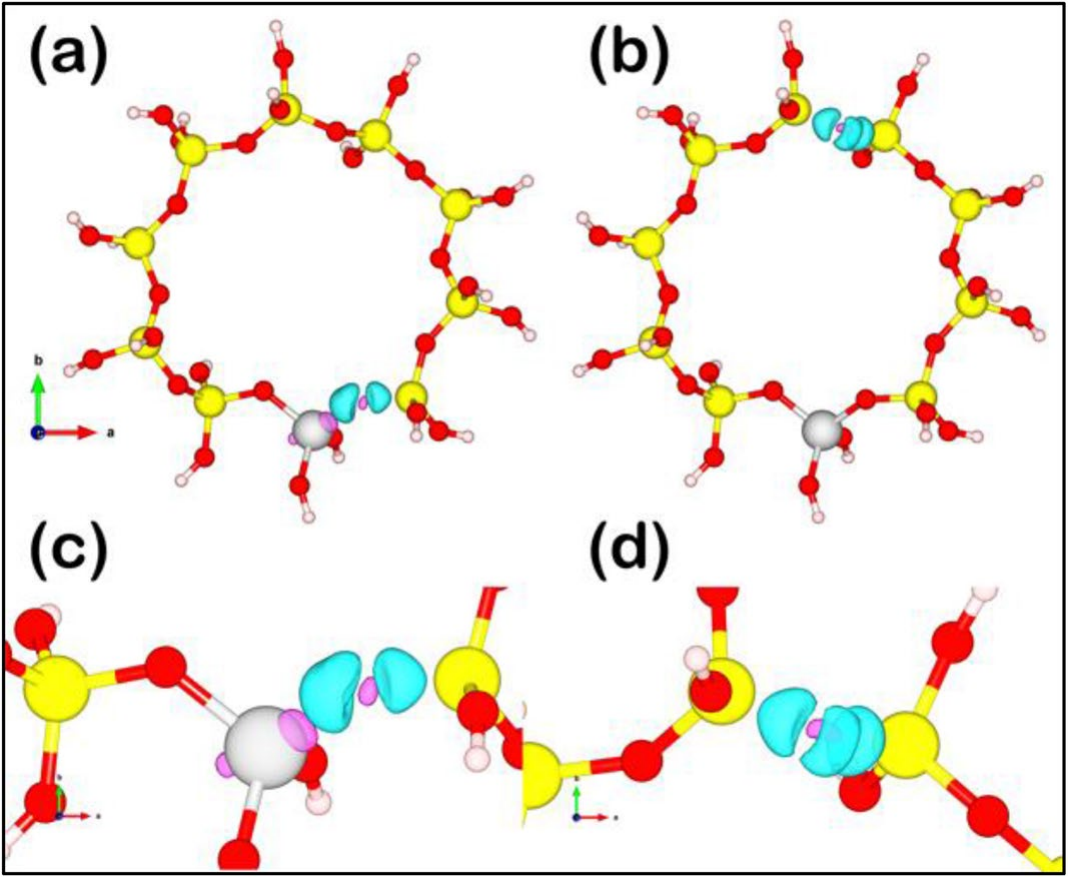
Electron transfer in terms of charge accumulation/depletion regions in TS-1 surface with (**a**, **c**) oxygen vacancy near the Ti-site (O_V,Ti_) and (**b**, **d**) oxygen vacancy far from the Ti-site (O_V,Si_). The blue regions represent charge accumulation, while the purple regions represent charge depletion.

For the TS-1 catalyst, two models of the deactivated surface were constructed: (1) TS-1 with O_V_ adjacent to the Ti-site (labeled as O_V,Ti_) and (2) TS-1 with O_V_ far from the Ti-site (labeled as O_V,Si_) illustrated in Fig. 6. The analysis of charge transfer in Table 2 suggested that only the location of O_V_ near the Ti-site affects the charge transfer of the Ti atom, whereas the O_V_ located far from the Ti-site affected the charge transfer of Si atoms near such O_V_ but not the Ti-site.

**Table 2:** The calculated charge transfer of P25 and TS-1.

Surface type	Ti in top layer	O in top layer	Ti4	Ti16	
P25*					
Fresh	+ 19.10	− 14.28	+ 2.41	+ 2.36	
With O_V_	+ 18.28	− 13.35	+ 2.03	+ 1.99	

Surface type	All Si	All O	Ti	Si2	Si6
TS-1*					
Fresh	+ 28.66	− 44.94	+ 2.28	+ 3.17	+ 3.19
With O_V,Ti_	+ 27.03	− 43.29	+ 2.07	+ 3.18	+ 3.19
With O_V,Si_	+ 27.47	− 43.49	+ 2.28	+ 2.38	+ 2.40

* All labeled atoms correlated to the label in Fig. 4.

**Figure 7:**
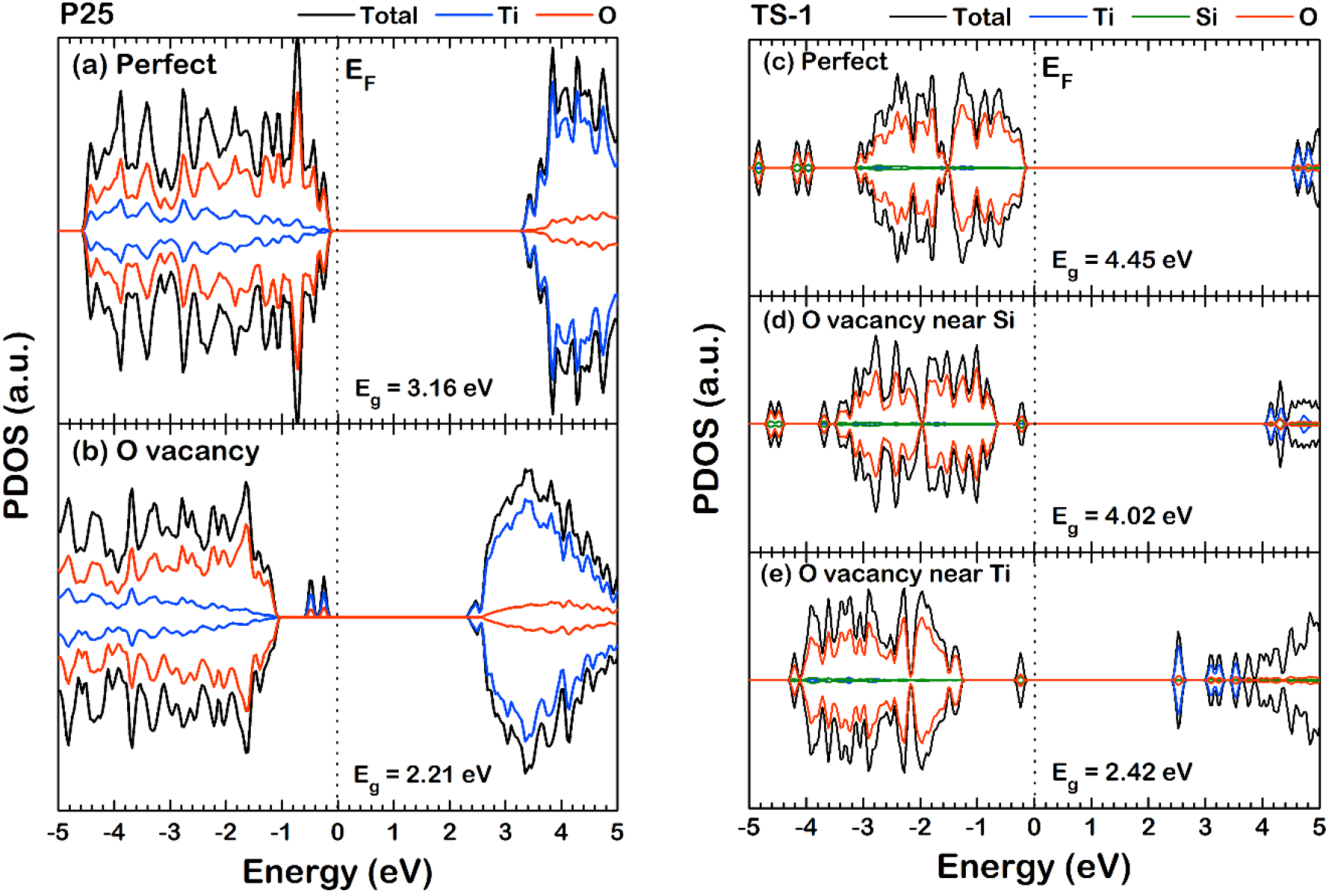
The projected density of state (PDOS) of (**a**) the fresh and (**b**) O vacancy for the P25 catalyst surface, and (**c**) the fresh, (**d**) the O_V,Si_, and (**e**) the O_V,Ti_ for TS-1 catalyst surface, respectively.

We further investigated the electronic effects of O_V_ on the surface apart from the charge transfer via the analysis of the projected density of state (PDOS). The spin-polarized PDOS of the fresh and deactivated surface of P25 and TS-1 was obtained and analyzed, as shown in Fig. 7, in which the Fermi level (E_F_) was set to 0 eV; thus, the negative and positive energy represents the valence state and conduction state, respectively. For the P25 catalyst, the energy gap (E_g_) incorporating the Hubbard U correction and D3 dispersion terms is 3.16 eV (DFT + *U* + D3, *U* = 6.00 eV), while Portillo-Vélez et al. ^27^ previously verified the E_g_ to be 2.98 eV with the Hubbard U correction but without the D3 dispersion term (DFT + *U*, *U* = 6.00 eV). As for this, we suggested that one should include the D3 term to represent well the P25; hence, our calculated band gap is reliable. On the PDOS profile of P25, the O-PDOS (red line) is the major contributor to the total density of state (TDOS) in the valence state, while the Ti-PDOS (blue line) is the main contribution in the TDOS of the conduction state. Analyzing the changes in PDOS of the P25 catalyst after the reaction via the comparison of the PDOS of fresh P25 and deactivated P25 is revealed that the E_g_ of the fresh surface of 3.16 eV reduced to 2.21 eV when the O_V_ formed. Also, on the deactivated surface, the conduction band minimum (CBM) shifted from 3.16 eV to 2.17 eV, while the valence band maximum (VBM) shifted from − 0.00089 eV to − 1.07 eV. Interestingly, with surface O_V_, unpaired electronic states were detected between − 0.1 eV and − 0.8 eV, comprised mainly of the Ti-state. These peaks correlated with the charge accumulation region confirmed via the Bader charge analysis, illustrated in Fig. 6.

For the TS-1, the calculated E_g_ is 4.45 eV (DFT + *U* + D3, *U* = 2.00 eV). The Hubbard *U* correction parameter for Ti in P25 is recommended to be 6 eV^27^, while for other oxide materials, in this case, TS-1, the *U* of 2.00 eV is suggested ^28^. Like in the case of P25, the Ti-PDOS is the major contributor in the conduction state, while O-PDOS is the major contributor in the valence state. The E_g_ value of the fresh surface at 4.45 eV reduced to 4.02 eV and 2.42 eV for the surface with O_V,Si_ and O_V,Ti_, respectively. The changes of CBM and VBM in the deactivated TS-1 are also similar to that of P25. The CBM shifted from 4.41 eV to 3.92 eV in the case of O_V,Si_ and shifted to 2.38 eV in O_V,Ti_, while, the VBM shifted from − 0.035 eV to − 0.556 eV in the case of O_V,Si_ and shifted to − 1.19 eV in O_V,Ti_. However, unlike the P25 surface with O_V_, the unpaired electronic states were found. The paired electronic state was detected in the valence state between − 0.0006 eV and − 0.417 eV for the TS-1 with O_V,Si_, while the one with O_V,Ti_ such state, is located between − 0.035 eV and − 0.42 eV. Both of the paired electronic states comprised the Si, Ti, and O states equally. Besides, in the conduction state, an interstate was also found at 2.5 eV only for the TS-1 with O_V,Ti_.

**Figure 8:**
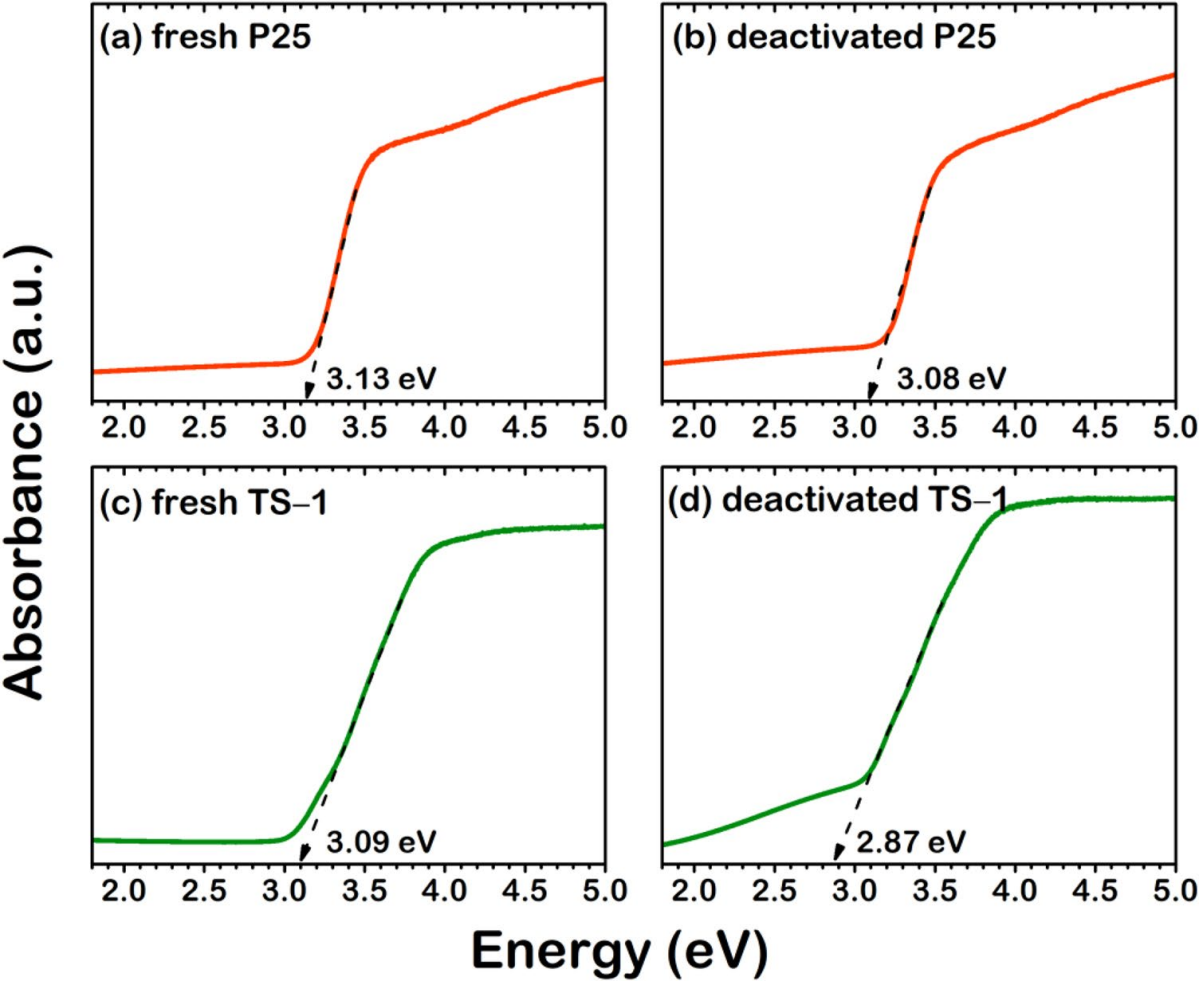
UV–vis spectrometry profile of (**a**) fresh P25 surface, (**b**) deactivated P25 surface, (**c**) fresh TS-1 surface, and (**d**) deactivated TS-1 surface.

As the calculated PDOS, which is indicated that the O_V_ induced the formation of the interstate leading to decrease E_g_, the UV–vis profiles of the fresh and spent catalysts are obtained and analyzed, shown in Fig. 8, in order to confirm these phenomena. The UV–vis profiles plotted the absorbance against energy were used to investigate the E_g_ for each sample. It is revealed that both spent P25 and TS-1 surface exhibited a lower E_g_ than their fresh surface. In addition, the E_g_ of TS-1 surface, which has a higher initial rate of deactivation, reduced more than that of the P25 surface.

Referring to the hypothesis for the deactivation mechanism, the evidence from the characterizations of the surface electronic properties of the fresh and spent P25 and TS-1 revealed the O_V_ is one of the causes altering the activity of the active region on the catalyst surface in addition to the fouling which can be handled via the calcination in air. This deactivation via O_V_ occurred through not only the changes in charge transfer, which promoted more Ti^3+^ species on the surface but also through the formation of the interstates found in the PDOS profile that altered the ability of the active region of the catalyst surface. The direct effect causing to lower conversion is the Ti^3+^ induced high binding energy between the active site, Ti–OOH, and MO. It can be implied that charge accumulation makes bonding of Ti–OOH strong. Therefore, the conversion of ME, which is formed by one O atom form Ti–OOH and MO, is significantly decreased.

## Conclusion

The following summarizes the deactivation mechanism of the P25 and TS-1 Ti-based oxide catalysts during the MO epoxidation.

(i) The fouling causes catalyst deactivation but in a regenerative way as the activity of the catalyst can be restored via the calcination in air oxidizing all the fouling species.
(ii) The catalyst surface also deactivated through the loss of oxygen atom, forming the surface O_V_ which induced the formation of the interstates in conduction and valence states of the catalyst, where these interstates resulted in the decrease in energy gap for both catalysts leading to the modification of the activity of the active region on the surface.
(iii) The O_V_ formation introduces a new electron-rich site on the deactivated surface in both P25, and TS-1 confirmed via the Bader charge analysis, where the charge accumulation is observed in the O_V_ site.

Therefore, as the cause of the reduced activity stemmed from the formation of the O_V_, the addition of the oxygen atom back to the same removal site would restore the activity during the reaction. However, during the reaction when the O atom was removed, the surface may have transformed into a more stable surface configuration prior to the addition of the oxygen atom via calcination. This way, the addition of the oxygen atom back to the removed site would not be possible, resulting in the permanent loss of the active region on the catalyst surface.

Ultimately, it was proposed that the cause of deactivation in Ti-based oxide catalysts during MO epoxidation is the combination of fouling and O_V_ formation. The fouling causes temporary deactivation and can be cured via calcination that oxidized the fouling species, while the O_V_ formation resulted in the surface modification that reduced the activity of the whole catalyst by altering the electronic properties of the active region on the surface producing a number of less active regions.

## Methodology

### Experimental details

#### Catalyst synthesis

The P25 catalyst was used the commercial TiO_2_, powder from Aeoxide (formerly Degussa), while the TS-1 catalyst was prepared by the hydrolysis of Ti(IV) isopropoxide (TIP, 97%, Sigma Aldrich) as Ti precursor and tetraethyl orthosilicate (TEOS, > 99%, Merck) as Si precursor by ammonium hydroxide (NH_4_OH, 28% solution, Sigma Aldrich). The isopropanol was used as the solvent in order to dissolve TIP in deionized water (DI water) prior to the transfer to autoclave. The synthetic gel formed with a molar ratio of 1.0TiO_2_:7.4SiO_2_:6.2(NH_4_)_2_O:1.8TPABr:553.7H_2_O in the 100 cm^3^ PTFE autoclave was heated at 1 °C/min to 160 °C and held for 2 h prior to the second heating to 210 °C at 10 °C/min for 5 h. The synthesized crystal was filtered, washed with DI water, and dried in air at 383 K overnight. The obtained solid was calcined at 550 °C for 6 h in air.

#### Catalyst characterization

The P25 and TS-1 catalysts were characterized by X-ray diffractometer (Bruker D8 Advance) using Cu Kα radiation with 2θ between 20° to 80° for P25 and 5° to 50° for TS-1, respectively, with a scan speed of 0.5 s/step. The elemental contents of Ti and O were analyzed by the X-ray photoelectron spectroscopy (XPS), where the spectra were obtained by AMICUS spectrometer using Mg Kα X-ray radiation (1253.6 eV) and Al Kα X-ray radiation (1486.6 eV) at a voltage of 15 kV and current of 12 mA. The energy gap for each catalyst was derived by the absorbance versus energy profiles obtained by the Ultraviolet–visible spectrophotometry (UV–vis), in which the absorbance from 200 to 500 nm is obtained by Perkin Elmer Lambda 650 spectrophotometer, where the step size for the scan is 1 nm.

#### Catalytic reaction testing

The catalytic property of each catalyst was carried out in a 50 cm^3^ three-necked round-bottom glass reactor with a reflux condenser. During the liquid-phase MO epoxidation reaction at 50 °C, the mixture was stirred with a magnetic stirrer at 500 rpm with a reaction of 5 h. The substrates comprise methyl oleate (MO, ˃99%, Sigma-Aldrich) and hydrogen peroxide (H_2_O_2_, 30 wt.% in H_2_O, Sigma-Aldrich) as an oxidizing reagent and acetonitrile (CH_3_CN, 99.8%, Sigma-Aldrich) as the solvent. The MO/H_2_O_2_ feed molar ratio is 3:1, where the naphthalene was used as an internal standard (C_10_H_8_, ˃98%, Fluka). In addition, the catalyst weighed 0.3 g, was mixed with MO before added with H_2_O_2_. After each batch of reaction, the catalyst will be collected and reused to analyze the stability over time. The recovering process entailed separation via centrifugation, washed with DI water, and dried overnight at 110 °C. Note that, in the studying of regeneration, the samples were further calcined in air at 550 °C for 5 h, but apart from that, all reused catalysts were followed the recovery step up to drying only. The product distribution for each run was analyzed by gas chromatography–mass spectrometry (GC–MS) equipped with a DB-5 column with a dimension of 30 m × 0.25 mm × 0.25 μm from Shimadzu.

#### Computational details

The DFT with the projector augmented wave (PAW) implemented in the Vienna ab initio simulation package (VASP) ^29–32^ was used to investigate the surface structure and electronic properties. The exchange–correlation functional along with the generalized gradient approximation (GGA) by Perdew, Burke, and Ernzerhof (PBE), especially the GGA + *U* was used ^33^. The parameter *U* of 6.0 and 2.0 was used for Ti in P25 ^27^ and TS-1 ^28^, respectively. The cut-off energy of 400 eV for P25 and 500 eV for TS-1 were used for the self-consistent loop. The structural optimization (OPT) was performed within the conjugate gradient method ^34^ and relaxed until the force convergence less than 0.01 eV/Å. Also, the Van der Waals dispersion term, DFT-D3 proposed by Grimme et al*.* ^35^ was applied for all calculations. In the Monkhorst–Pack *k*-mesh Brillouin-zone integration ^36^, the 3 × 3 × 1 and 3 × 3 × 3 were used for P25 and TS-1 during the OPT. In the density of state (DOS) calculation, the value of 5 × 5 × 1 and 5 × 5 × 5 was used for P25 and TS-1. All structure models were drawn and visualized by VESTA package ^37^.

The partial charge accumulation or depletion during O vacancy formation (${{\Delta \updelta }}_{{\mathrm{O- vac}}}$) on the surfaces was calculated based on the Bader charge analysis ^38–41^ as follows:

1 $${{\Delta \updelta }}_{{\text{O-vac}}} = \updelta_{{\mathrm{O-vac,Sur}}} {{{-} \updelta }}_{{\mathrm{Fresh,Sur}}} + {\updelta }_{{\mathrm{O}}}$$

The parameters ${\updelta }_{{\mathrm{O - vac,Sur}}}$, ${\updelta }_{{\mathrm{Fresh,Sur}}}$, and ${\updelta }_{{\mathrm{O}}}$ are the partial charge of the surface with O vacancy, clean surface, and an O atom, respectively. Additionally, the methodology of designed models is revealed in the Results and Discussion section.

## Supplementary information

Supplementary Information

## Data Availability

The authors declare that relevant data are within the manuscript.
