# Supplementary material for: Performance controlled via surface oxygen-vacancy in Ti-based oxide catalyst during methyl oleate epoxidation
Source: Sci Rep. 2020 Nov 3;10:18952. doi: 10.1038/s41598-020-76094-2 (PMC7641232; doi:10.1038/s41598-020-76094-2)
Supplement: Supplementary file 1 — Supplementary Information [file 41598_2020_76094_MOESM1_ESM.pdf]

Supplementary document

Performance controlled via surface oxygen-vacancy in Ti-based oxide catalyst during methyl oleate epoxidation

Supareak Praserthdam[1,2], Meena Rittiruam[1,2], Kanokpon Maungthong[2], Tinnakorn Saelee[1,2], Siriwimol Somdee[1,2], Piyasan Praserthdam[2,*]

[1] High-Performance Computing Unit (CECC-HCU), Center of Excellence on Catalysis and Catalytic Reaction Engineering (CECC), Chulalongkorn University, Bangkok 10330 Thailand

[2] Center of Excellence on Catalysis and Catalytic Reaction Engineering (CECC), Chulalongkorn University, Bangkok 10330 Thailand

*Corresponding author's email: piyasan.p@chula.ac.th (Prof. Piyasan Praserthdam)

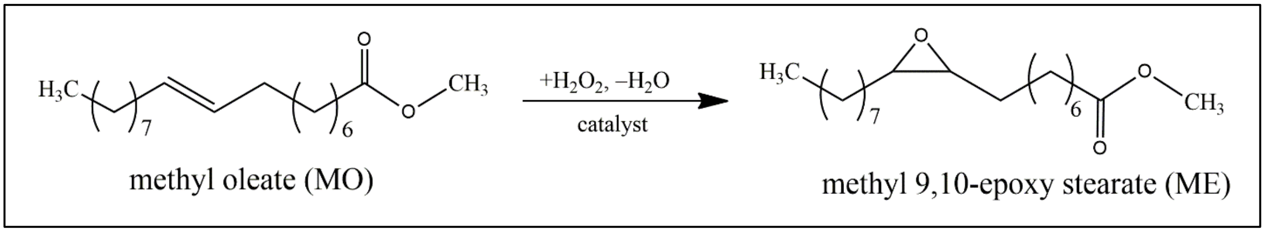

Figure S1. The proposed methyl oleate epoxidation reaction with H_2O_2, redrawn base on Reference [1].

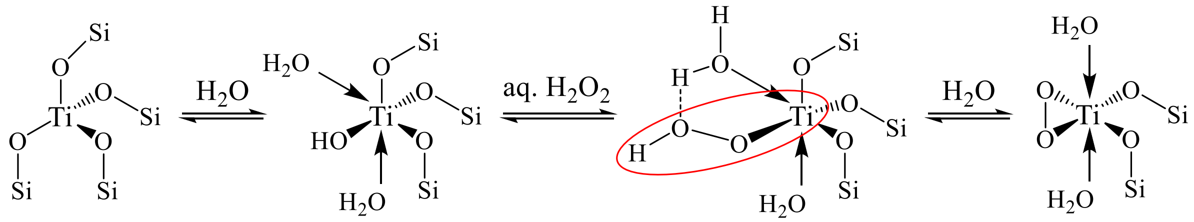

Figure S2. Mechanism of inactivation of amorphous titanosilicate epoxidation catalysts, redrawn base on Reference[2]

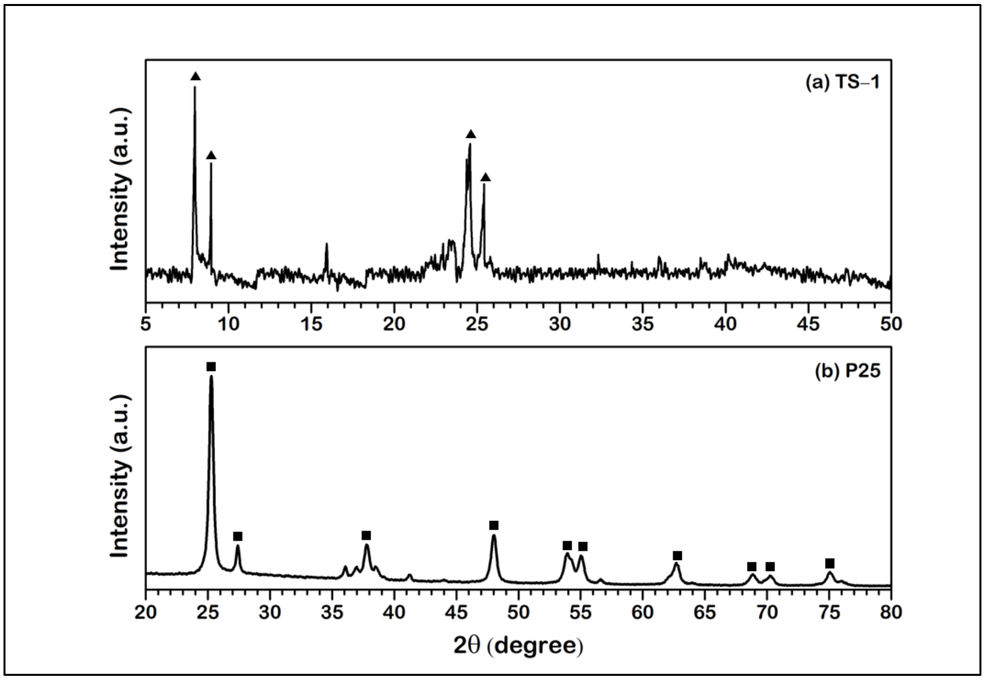

Figure S3 The XRD patterns of (a) TS-1 and (b) P25 catalysts.

XRD patterns as shown in Figure S1 is the TS–1 and P25 to confirm the phase structure. For P25, we found the diffraction peaks at 25.2º, 37.8º, 48.0º, 53.8º, 55.0º and 62.6º that can be defined to the (101), (004), (200), (105), (211), and (204) diffraction plane of anatase TiO_2 that agrees with Reference [3]. For TS-1, we found the diffraction peaks at 7.9º, 8.9º, 23.2º, 23.9º, and 24.4º that can be defined to the MFI structure of highly crystalline TS–1 or ZSM–5 that agrees with Reference [4]. Consequently, we specify the phase of P25 as the anatase TiO_2 and the phase of TS–1 as the ZSM–5.

Table S1. XPS data of different oxidation state of Ti and O on the surface of TiO_2 (P25) and titanosilicate (TS-1) where O_L represents the lattice oxygen, O_C represents the sub oxide, and O_V represents the oxygen vacancies.

Catalyst		Ti^{4+} (unit area)		Total $\%Ti^{4+}$	Ti^{3+} (unit area)		Total $\%Ti^{3+}$	O (unit area)			Total $\%O_V$
		Ti2p3/2	Ti2p1/2		Ti2p3/2	Ti2p1/2		O_L	O_C	O_V	
P25	before	18,686.4	-	86.7	-	2,865.2	13.3	13,214.5	12,103.1	-	-
	after	7,307.5	-	85.9	-	1,206.7	14.2	4,770.2	7,719.4	3,239.2	20.6
TS-1	before	3,557.4	-	91.7	-	321.6	8.3	5,397.5	55,759.8	-	-
	after	3,163.2	-	81.1	-	1,210.0	18.9	1,140.0	17,755.0	5,093.9	21.2

Structural parameter: anatase TiO_2 bulk structure

```
TiO2
1.0
        3.8061339900         0.0000000000         0.0000000000
        0.0000000000         3.8061339000         0.0000000000
        0.0000000000         0.0000000000         9.7243557000
   Ti    O
    4    8
Direct
     0.500000000         0.500000000         0.000000000
     0.500000000        -0.000000000         0.250000000
    -0.000000000        -0.000000000         0.500000000
    -0.000000000         0.500000000         0.750000000
    -0.000000000         0.500000000         0.956412970
     0.500000000         0.500000000         0.206412995
     0.500000000         0.000000000         0.043586999
    -0.000000000        -0.000000000         0.293587005
     0.500000000        -0.000000000         0.456413019
    -0.000000000        -0.000000000         0.706412970
     0.000000000         0.500000000         0.543587030
     0.500000000         0.500000000         0.793587030
```

Structural parameter: $Si_{80}Ti_{16}O_{192}$ **bulk structure**

```
Si80Ti16O192
1.0
      20.0699996948         0.0000000000         0.0000000000
       0.0000000000        19.9200000763         0.0000000000
       0.0000000000         0.0000000000        13.4200000763
   Si   Ti    O
   80   16  192
Direct
     0.423199993         0.060500001         0.669399988
     0.576800007         0.939499993         0.330599976
     0.076800001         0.939499993         0.169400006
     0.923199993         0.060500001         0.830600012
     0.576800007         0.560500007         0.330599976
     0.423199993         0.439499993         0.669399988
     0.923199993         0.439499993         0.830600012
     0.076800001         0.560500007         0.169400006
     0.690999966         0.528100023         0.184900003
     0.308999987         0.471899977         0.815100033
     0.808999987         0.471899977         0.684899967
     0.191000001         0.528100023         0.315099997
     0.278800014         0.060199997         0.031200000
     0.721199986         0.939799979         0.968799998
     0.221200009         0.939799979         0.531200002
     0.778800014         0.060199997         0.468799998
     0.721199986         0.560199973         0.968799998
     0.278800014         0.439799979         0.031200000
     0.778800014         0.439799979         0.468799998
     0.221200009         0.560199973         0.531200002
     0.122000000         0.064099996         0.032600001
     0.878000011         0.935899968         0.967400044
     0.377999988         0.935899968         0.532599992
     0.621999989         0.064099996         0.467400008
     0.878000011         0.564100032         0.967400044
     0.122000000         0.435900016         0.032600001
     0.621999989         0.435900016         0.467400008
     0.377999988         0.564100032         0.532599992
     0.927200008         0.527800037         0.182199992
     0.072800004         0.472200011         0.817799954
     0.572799992         0.472200011         0.682200046
     0.427200008         0.527800037         0.317799990
     0.188400003         0.057300003         0.675100004
     0.811600044         0.942700068         0.324899996
     0.311599997         0.942700068         0.175100004
     0.688399956         0.057300003         0.824899996
     0.811600044         0.557299979         0.324899996
     0.188400003         0.442700021         0.675100004
     0.688399956         0.442700021         0.824899996
     0.311599997         0.557299979         0.175100004
     0.307599981         0.871399987         0.815199948
     0.692399971         0.128600001         0.184799998
     0.192399995         0.128600001         0.315199984
     0.807600029         0.871399987         0.684800052
     0.692399971         0.371399987         0.184799998
     0.307599981         0.628600013         0.815199948
     0.807600029         0.628600013         0.684800052
     0.192399995         0.371399987         0.315199984
     0.274799999         0.827799932         0.036600001
     0.725200001         0.172199996         0.963400012
     0.225200001         0.172199996         0.536599988
     0.774799999         0.827799932         0.463400012
```

0.725200001	0.327800004	0.963400012
0.274799999	0.672200020	0.036600001
0.774799999	0.672200020	0.463400012
0.225200001	0.327800004	0.536599988
0.119900004	0.827099996	0.026900000
0.880099996	0.172900004	0.973099989
0.380100019	0.172900004	0.526899976
0.619900004	0.827099996	0.473100024
0.880099996	0.327100020	0.973099989
0.119900004	0.672900004	0.026900000
0.619900004	0.672900004	0.473100024
0.380100019	0.327100020	0.526899976
0.070200006	0.869599975	0.817400008
0.929799971	0.130400001	0.182600010
0.429800018	0.130400001	0.317400008
0.570200029	0.869599975	0.682599992
0.929799971	0.369599999	0.182600010
0.070200006	0.630400025	0.817400008
0.570200029	0.630400025	0.682599992
0.429800018	0.369599999	0.317400008
0.189400007	0.825799992	0.682799965
0.810599993	0.174199996	0.317199999
0.310600017	0.174199996	0.182800001
0.689400007	0.825799992	0.817200035
0.810599993	0.325799992	0.317199999
0.189400007	0.674200008	0.682799965
0.689400007	0.674200008	0.817200035
0.310600017	0.325799992	0.182800001
0.308999987	0.028099999	0.815100033
0.690999966	0.971900025	0.184900003
0.191000001	0.971900025	0.315099997
0.808999987	0.028099999	0.684899967
0.072800004	0.027799998	0.817799954
0.927200008	0.972200011	0.182199992
0.427200008	0.972200011	0.317799990
0.572799992	0.027799998	0.682200046
0.423199993	0.830499998	0.674199983
0.576800007	0.169499990	0.325800017
0.076800001	0.169499990	0.174200001
0.923199993	0.830499998	0.825800017
0.576800007	0.330499998	0.325800017
0.423199993	0.669500002	0.674199983
0.923199993	0.669500002	0.825800017
0.076800001	0.330499998	0.174200001
0.372799992	0.066499998	0.760300002
0.627200008	0.933499984	0.239699998
0.127200008	0.933499984	0.260300002
0.872799992	0.066499998	0.739699998
0.627200008	0.566500016	0.239699998
0.372799992	0.433499984	0.760300002
0.872799992	0.433499984	0.739699998
0.127200008	0.566500016	0.260300002
0.309500012	0.062100003	0.923899988
0.690500035	0.937900003	0.076100003
0.190500011	0.937900003	0.423900023
0.809499965	0.062100003	0.576099977
0.690500035	0.562099997	0.076100003
0.309500012	0.437900003	0.923899988
0.809499965	0.437900003	0.576099977
0.190500011	0.562099997	0.423900023
0.199300009	0.060500001	0.026300001
0.800700026	0.939499993	0.973699979

0.300700003	0.939499993	0.526300021
0.699299974	0.060500001	0.473699979
0.800700026	0.560500007	0.973699979
0.199300009	0.439499993	0.026300001
0.699299974	0.439499993	0.473699979
0.300700003	0.560500007	0.526300021
0.100000000	0.057099999	0.918299958
0.900000000	0.942899995	0.081699997
0.400000000	0.942899995	0.418299994
0.600000048	0.057099999	0.581700042
0.900000000	0.557100005	0.081699997
0.100000000	0.442899995	0.918299958
0.600000048	0.442899995	0.581700042
0.400000000	0.557100005	0.418299994
0.118299998	0.059199997	0.730299981
0.881700002	0.940799997	0.269699983
0.381700002	0.940799997	0.230299999
0.618299998	0.059199997	0.769700019
0.881700002	0.559200003	0.269699983
0.118299998	0.440799997	0.730299981
0.618299998	0.440799997	0.769700019
0.381700002	0.559200003	0.230299999
0.240700012	0.043999999	0.758200000
0.759300012	0.955999995	0.241800000
0.259299988	0.955999995	0.258199982
0.740699988	0.043999999	0.741800000
0.759300012	0.544000053	0.241800000
0.240700012	0.455999995	0.758200000
0.740699988	0.455999995	0.741800000
0.259299988	0.544000053	0.258199982
0.375000000	0.849099999	0.763900016
0.625000000	0.150900001	0.236100001
0.125000000	0.150900001	0.263900016
0.874999952	0.849099999	0.736099984
0.625000000	0.349099999	0.236100001
0.375000000	0.650900001	0.763900016
0.874999952	0.650900001	0.736099984
0.125000000	0.349099999	0.263900016
0.307900006	0.848599990	0.931900050
0.692099994	0.151399998	0.068099998
0.192100006	0.151399998	0.431899979
0.807900054	0.848599990	0.568099985
0.692099994	0.348599990	0.068099998
0.307900006	0.651400010	0.931900050
0.807900054	0.651400010	0.568099985
0.192100006	0.348599990	0.431899979
0.198100005	0.845900019	0.032400001
0.801900031	0.154100005	0.967600017
0.301900007	0.154100005	0.532400019
0.698099969	0.845900019	0.467599981
0.801900031	0.345899995	0.967600017
0.198100005	0.654099981	0.032400001
0.698099969	0.654099981	0.467599981
0.301900007	0.345899995	0.532400019
0.090899995	0.834799959	0.924899996
0.909099963	0.165199993	0.075099995
0.409100011	0.165199993	0.424899996
0.590899989	0.834799959	0.575100004
0.909099963	0.334800007	0.075099995
0.090899995	0.665199993	0.924899996
0.590899989	0.665199993	0.575100004
0.409100011	0.334800007	0.424899996

0.123000004	0.857299970	0.736800032
0.876999960	0.142700006	0.263200021
0.377000008	0.142700006	0.236799996
0.623000040	0.857299970	0.763199968
0.876999960	0.357300018	0.263200021
0.123000004	0.642700030	0.736800032
0.623000040	0.642700030	0.763199968
0.377000008	0.357300018	0.236799996
0.252900011	0.833999964	0.760700020
0.747100013	0.165999988	0.239299998
0.247099989	0.165999988	0.260699984
0.752899987	0.833999964	0.739299980
0.747100013	0.333999988	0.239299998
0.252900011	0.665999988	0.760700020
0.752899987	0.665999988	0.739299980
0.247099989	0.333999988	0.260699984
0.311199995	0.948100010	0.817700039
0.688799957	0.051900002	0.182299997
0.188800005	0.051900002	0.317700003
0.811200043	0.948100010	0.682299961
0.688799957	0.448100010	0.182299997
0.311199995	0.551900038	0.817700039
0.811200043	0.551900038	0.682299961
0.188800005	0.448100010	0.317700003
0.079000003	0.947399978	0.825699959
0.921000032	0.052599998	0.174300005
0.420999985	0.052599998	0.325700030
0.579000015	0.947399978	0.674300041
0.921000032	0.447400026	0.174300005
0.079000003	0.552600022	0.825699959
0.579000015	0.552600022	0.674300041
0.420999985	0.447400026	0.325700030
0.409100011	0.125799995	0.612999959
0.590899989	0.874200017	0.387000005
0.090899995	0.874200017	0.112999995
0.909099963	0.125799995	0.887000041
0.590899989	0.625800031	0.387000005
0.409100011	0.374199993	0.612999959
0.909099963	0.374199993	0.887000041
0.090899995	0.625800031	0.112999995
0.416000013	0.001100000	0.582299997
0.583999987	0.998900019	0.417700003
0.083999999	0.998900019	0.082299997
0.915999966	0.001100000	0.917699968
0.583999987	0.501099981	0.417700003
0.416000013	0.498900019	0.582299997
0.915999966	0.498900019	0.917699968
0.083999999	0.501099981	0.082299997
0.397599991	0.870000020	0.580900007
0.602400009	0.129999992	0.419099993
0.102399997	0.129999992	0.080899998
0.897599991	0.870000020	0.919099993
0.602400009	0.369999996	0.419099993
0.397599991	0.629999980	0.580900007
0.897599991	0.629999980	0.919099993
0.102399997	0.369999996	0.080899998
0.184700001	0.129500007	0.617600052
0.815300034	0.870500029	0.382400019
0.315299987	0.870500029	0.117599999
0.684700013	0.129500007	0.882399948
0.815300034	0.629499971	0.382400019
0.184700001	0.370500005	0.617600052

0.684700013	0.370500005	0.882399948
0.315299987	0.629499971	0.117599999
0.203700014	0.003000000	0.597999984
0.796300010	0.996999947	0.402000016
0.296299986	0.996999947	0.097999993
0.703699990	0.003000000	0.902000016
0.796300010	0.503000005	0.402000016
0.203700014	0.496999995	0.597999984
0.703699990	0.496999995	0.902000016
0.296299986	0.503000005	0.097999993
0.191000001	0.874100054	0.585300020
0.808999987	0.125900006	0.414700015
0.308999987	0.125900006	0.085300002
0.690999966	0.874100054	0.914699944
0.808999987	0.374100006	0.414700015
0.191000001	0.625899994	0.585300020
0.690999966	0.625899994	0.914699944
0.308999987	0.374100006	0.085300002
0.998399994	0.042500000	0.794399970
0.001600000	0.957500021	0.205599994
0.501600006	0.957500021	0.294400006
0.498399994	0.042500000	0.705600030
0.001600000	0.542500027	0.205599994
0.998399994	0.457500021	0.794399970
0.498399994	0.457500021	0.705600030
0.501600006	0.542500027	0.294400006
0.998399994	0.843500035	0.792200053
0.001600000	0.156500001	0.207800001
0.501600006	0.156500001	0.292199999
0.498399994	0.843500035	0.707799947
0.001600000	0.343499987	0.207800001
0.998399994	0.656499965	0.792200053
0.498399994	0.656499965	0.707799947
0.501600006	0.343499987	0.292199999
0.427700034	0.750000024	0.649700005
0.572300013	0.250000000	0.350300031
0.072300002	0.250000000	0.149700005
0.927699987	0.750000024	0.850299995
0.204499993	0.750000024	0.652399997
0.795499959	0.250000000	0.347600003
0.295500007	0.250000000	0.152399997
0.704500041	0.750000024	0.847600003
0.286099995	0.750000024	0.058999999
0.713900029	0.250000000	0.940999965
0.213900005	0.250000000	0.558999999
0.786099971	0.750000024	0.441000001
0.108899998	0.750000024	0.058000000
0.891099990	0.250000000	0.941999973
0.391099990	0.250000000	0.558000027
0.608900010	0.750000024	0.442000009

Structural parameter: anatase TiO_2-101 surface model (P25)

```
P25
   1.0000000000000000
    11.1524000167999997    0.0000000000000000    0.0000000000000000
    -2.5920273348000000    7.1481555410000004    0.0000000000000000
     0.0000000000000000    0.0000000000000000   18.9027004241999990
   Ti   O
    16    32
Selective dynamics
Direct
  0.0230808535087684  0.0114937440261727  0.2366150608077055   T   T   T
  0.0230808545087650  0.5114937940261766  0.2366150608077055   T   T   T
  0.5231016916504652  0.0115017496229254  0.2366225612215605   T   T   T
  0.5231016876504648  0.5115017996229294  0.2366225612215605   T   T   T
  0.3799299829999967  0.4399700000000024  0.0496199990000008   F   F   F
  0.3799299710000028  0.9399700100000032  0.0496199990000008   F   F   F
  0.8799300259999967  0.4399700000000024  0.0496199990000008   F   F   F
  0.8799300129999992  0.9399700100000032  0.0496199990000008   F   F   F
  0.1498399999999975  0.0749000030000033  0.0739400040000007   F   F   F
  0.1498400010000012  0.5749000050000035  0.0739400040000007   F   F   F
  0.6498400219999994  0.0749000030000033  0.0739400040000007   F   F   F
  0.6498400070000017  0.5749000050000035  0.0739400040000007   F   F   F
  0.2910217413612758  0.1456316295383838  0.2656614266557690   T   T   T
  0.2910217323612715  0.6456316065383784  0.2656614266557690   T   T   T
  0.7909391708523659  0.1455745272009180  0.2656678076659504   T   T   T
  0.7909391508523642  0.6455745042009127  0.2656678076659504   T   T   T
  0.1903499880000012  0.3451500119999977  0.0742599940000019   F   F   F
  0.1903499829999973  0.8451499889999994  0.0742599940000019   F   F   F
  0.6903399830000012  0.3451500119999977  0.0742599940000019   F   F   F
  0.6903399629999996  0.8451499889999994  0.0742599940000019   F   F   F
  0.3395178243025358  0.4198486246024544  0.2791691638782784   T   T   T
  0.3395178333025401  0.9198486346024553  0.2791691638782784   T   T   T
  0.8395327889375385  0.4198424369421660  0.2791707829617417   T   T   T
  0.8395328189375409  0.9198424469421669  0.2791707829617417   T   T   T
  0.4693399889999981  0.4846800150000021  0.1296499990000015   F   F   F
  0.4693399679999999  0.9846799919999967  0.1296499990000015   F   F   F
  0.9693400310000015  0.4846800150000021  0.1296499990000015   F   F   F
  0.9693400109999999  0.9846799919999967  0.1296499990000015   F   F   F
  0.1355068743256835  0.0678735032288004  0.3138838936598996   T   T   T
  0.1355068713256868  0.5678735392288033  0.3138838936598996   T   T   T
  0.6355343337307527  0.0678870021692845  0.3138738444023185   T   T   T
  0.6355343277307522  0.5678870381692875  0.3138738444023185   T   T   T
  0.3294399749999997  0.1647299950000018  0.0360999990000010   F   F   F
  0.3294399879999972  0.6647300220000005  0.0360999990000010   F   F   F
  0.8294400170000031  0.1647299950000018  0.0360999990000010   F   F   F
  0.8294400090000025  0.6647300220000005  0.0360999990000010   F   F   F
  0.4778687743991269  0.2389413436860494  0.2374314925438752   T   T   T
  0.4778687663991263  0.7389413706860481  0.2374314925438752   T   T   T
  0.9778837060938997  0.2389473871176515  0.2374320333898487   T   T   T
  0.9778836980938991  0.7389474141176505  0.2374320333898487   T   T   T
  0.0340000030000027  0.0169900010000035  0.0012100000000004   F   F   F
  0.0340000070000031  0.5169900329999990  0.0012100000000004   F   F   F
  0.5339999600000027  0.0169900010000035  0.0012100000000004   F   F   F
  0.5339999959999986  0.5169900329999990  0.0012100000000004   F   F   F
  0.1923077330235979  0.0962226062036662  0.1830273909299759   T   T   T
  0.1923077310235977  0.5962225912036683  0.1830273909299759   T   T   T
  0.6923261238902970  0.0962317823581366  0.1830408359702363   T   T   T
  0.6923261058903026  0.5962317673581389  0.1830408359702363   T   T   T
```

Structural parameter: anatase TiO_2-101 surface model (P25) with O vacancy

```
P25-O-vacancy
   1.0000000000000000
    11.1524000167999997    0.0000000000000000    0.0000000000000000
    -2.5920273348000000    7.1481555410000004    0.0000000000000000
     0.0000000000000000    0.0000000000000000   18.9027004241999990
   Ti   O
    16    31
Selective dynamics
Direct
  0.0148067607267706  0.0094117326570284  0.2370475796199258   T   T   T
  0.0147563012477931  0.5052197371185656  0.2370403966815076   T   T   T
  0.5162072950565033  0.0082704302278316  0.2388557198738042   T   T   T
  0.5039164546746093  0.5021764244017817  0.2315255965571410   T   T   T
  0.3799310010000028  0.4399726349999966  0.0496157299999993   F   F   F
  0.3799310079999998  0.9399726790000003  0.0496157299999993   F   F   F
  0.8799309799999975  0.4399726349999966  0.0496157299999993   F   F   F
  0.8799309750000006  0.9399726790000003  0.0496157299999993   F   F   F
  0.1498395389999985  0.0749030300000015  0.0739413149999990   F   F   F
  0.1498395320000014  0.5749030070000032  0.0739413149999990   F   F   F
  0.6498394850000011  0.0749030300000015  0.0739413149999990   F   F   F
  0.6498395079999995  0.5749030070000032  0.0739413149999990   F   F   F
  0.2813371896600927  0.1408051037794564  0.2653910075577599   T   T   T
  0.2812887267285262  0.6412941628186601  0.2653970861331700   T   T   T
  0.7862257154420647  0.1433911552084837  0.2647701200534627   T   T   T
  0.7958116741972532  0.6485226391294674  0.2592644195126823   T   T   T
  0.1903452669999979  0.3451532480000026  0.0742608139999987   F   F   F
  0.1903452700000017  0.8451532580000034  0.0742608139999987   F   F   F
  0.6903452669999979  0.3451532480000026  0.0742608139999987   F   F   F
  0.6903452549999969  0.8451532580000034  0.0742608139999987   F   F   F
  0.3229595258043361  0.4117812663272886  0.2780340270415849   T   T   T
  0.3302301872655065  0.9153698650593611  0.2749714224059332   T   T   T
  0.8378497288129907  0.4132502012877168  0.2824531940736748   T   T   T
  0.8378417170034140  0.9247521029023886  0.2827375719874254   T   T   T
  0.4693360380000016  0.4846768130000001  0.1296503270000002   F   F   F
  0.4693360540000029  0.9846768569999966  0.1296503270000002   F   F   F
  0.9693360380000016  0.4846768130000001  0.1296503270000002   F   F   F
  0.9693360329999976  0.9846768569999966  0.1296503270000002   F   F   F
  0.1285103556595928  0.0631628217441147  0.3145539975549525   T   T   T
  0.1284543164133183  0.5655928293065742  0.3145552317348148   T   T   T
  0.6324269061907870  0.0664443213437735  0.3148059736250062   T   T   T
  0.3294408820000001  0.1647280099999975  0.0360983369999985   F   F   F
  0.3294408909999973  0.6647280210000019  0.0360983369999985   F   F   F
  0.8294409239999965  0.1647280099999975  0.0360983369999985   F   F   F
  0.8294408690000026  0.6647280210000019  0.0360983369999985   F   F   F
  0.4768106819386517  0.2385406268440136  0.2488000680233355   T   T   T
  0.4768697253583483  0.7387193614200527  0.2488223984090965   T   T   T
  0.9732913150514477  0.2366072457704576  0.2334540128694442   T   T   T
  0.9797789978988902  0.7398993495817336  0.2341047840127217   T   T   T
  0.0339983100000012  0.0169921330000022  0.0012100479999972   F   F   F
  0.0339983109999977  0.5169921680000016  0.0012100479999972   F   F   F
  0.5339983180000019  0.0169921330000022  0.0012100479999972   F   F   F
  0.5339983109999977  0.5169921680000016  0.0012100479999972   F   F   F
  0.1820677019999977  0.0910100509999978  0.1825942420000004   F   F   F
  0.1820677019999977  0.5910100529999980  0.1825942420000004   F   F   F
  0.6820677130000021  0.0910100509999978  0.1825942420000004   F   F   F
  0.6820676990000010  0.5910100529999980  0.1825942420000004   F   F   F
```

Structural parameter: TS–1

```
TS–1
   1.0000000000000000
    25.0000000000000000    0.0000000000000000    0.0000000000000000
     0.0000000000000000   25.0000000000000000    0.0000000000000000
     0.0000000000000000    0.0000000000000000   15.0000000000000000
   Si   H    Ti   O
     9    20     1    30
Selective dynamics
Direct
  0.3125700000000009  0.4337699889999982  0.5648300169999985   F   F   F
  0.5758799740000029  0.6725399780000032  0.4627699850000013   F   F   F
  0.6687300110000010  0.5925999829999995  0.4696700100000015   F   F   F
  0.6146699909999995  0.3710499949999999  0.5463399890000034   F   F   F
  0.3649200059999984  0.6554100039999966  0.4966499960000021   F   F   F
  0.4727199940000020  0.7026000210000021  0.5624299999999991   F   F   F
  0.3899399949999989  0.3668700030000025  0.4476900100000023   F   F   F
  0.6749399569999994  0.4810299680000014  0.5460199990000021   F   F   F
  0.3041299819999992  0.5487800220000025  0.4893600150000026   F   F   F
  0.3010500139999976  0.4317300029999984  0.7233800249999973   F   F   F
  0.6934200290000021  0.5972300339999990  0.3082500140000022   F   F   F
  0.4615900040000014  0.7978800200000009  0.5187299729999992   F   F   F
  0.5423300169999976  0.6596099850000030  0.3138700169999993   F   F   F
  0.2982299999999967  0.6987799839999980  0.5894700369999981   F   F   F
  0.6815799709999979  0.3271900180000031  0.4498699820000027   F   F   F
  0.3559799960000021  0.6921499629999985  0.3459599810000000   F   F   F
  0.6195799640000033  0.7602199549999966  0.4624100050000024   F   F   F
  0.5334699630000017  0.3036200140000034  0.3035600030000012   F   F   F
  0.4585900120000019  0.7208100129999977  0.7225099560000032   F   F   F
  0.7499300380000022  0.6203799820000029  0.5577800109999984   F   F   F
  0.3815000150000003  0.4080199810000025  0.2991900130000005   F   F   F
  0.2292499919999997  0.5780900190000011  0.5842699689999975   F   F   F
  0.7528199770000015  0.4432099910000034  0.4639799750000009   F   F   F
  0.2750900079999994  0.5506200029999988  0.3303200089999976   F   F   F
  0.6227800369999983  0.3279899979999996  0.6942800520000034   F   F   F
  0.7030100249999975  0.4885799790000007  0.6994600299999973   F   F   F
  0.4992300030000010  0.2347900010000004  0.5228199960000026   F   F   F
  0.2251000019999978  0.4023600009999981  0.5072600359999981   F   F   F
  0.3529399869999992  0.2768000029999982  0.4394400279999999   F   F   F
  0.5030019855201849  0.3294356690166851  0.4537281145861695   T   T   T
  0.3208599849999985  0.4413999940000011  0.6718000409999973   F   F   F
  0.5498200230000023  0.6469799799999976  0.3723199840000007   F   F   F
  0.6216199870000025  0.6328899769999978  0.5030400280000009   F   F   F
  0.6749800110000024  0.6003699870000005  0.3633599919999995   F   F   F
  0.6485199740000027  0.3336400219999973  0.4791400269999997   F   F   F
  0.3323699950000005  0.6960099790000029  0.5620100020000010   F   F   F
  0.3521200180000008  0.6668099979999980  0.3926699960000022   F   F   F
  0.6016399769999978  0.7300099950000032  0.4383399960000034   F   F   F
  0.3280299760000034  0.4903699870000011  0.5165899910000036   F   F   F
  0.6492599489999975  0.5315600200000006  0.4920499799999973   F   F   F
  0.7230200199999999  0.6026800160000008  0.5241199809999983   F   F   F
  0.5310900119999999  0.6766699980000013  0.5413699469999997   F   F   F
  0.6308300020000033  0.4339999769999991  0.5371199929999975   F   F   F
  0.3488800049999981  0.5934200290000007  0.5173599880000026   F   F   F
  0.4663999939999997  0.7025900269999994  0.6685000100000025   F   F   F
  0.3741100310000007  0.4009700009999975  0.3601799959999994   F   F   F
  0.4511095476682295  0.3779301159291384  0.4750261475691472   T   T   T
  0.5190569140504725  0.3343543547972386  0.3342654409035536   T   T   T
  0.2476900099999995  0.5575400159999973  0.5407900490000017   F   F   F
  0.7317699430000033  0.4651199719999966  0.5019999820000010   F   F   F
  0.2962900160000004  0.5516999820000024  0.3828599929999967   F   F   F
```

0.4281400300000016	0.6632599639999981	0.5176500000000033	F	F	F
0.4702600099999970	0.7609000400000028	0.5168300309999978	F	F	F
0.5529439064138550	0.3610513022935681	0.5216595593523268	T	T	T
0.3540800090000005	0.3885599899999974	0.5311400410000005	F	F	F
0.6231600189999966	0.3545899960000014	0.6490999860000031	F	F	F
0.6822399899999994	0.4995299910000028	0.6496100110000000	F	F	F
0.2515999980000032	0.4157799910000008	0.5467299780000019	F	F	F
0.3796800229999988	0.3036400029999982	0.4347999889999983	F	F	F
0.4838596774354009	0.2618420319899410	0.4854250794639313	T	T	T

Structural parameter: TS–1 with O vacancy near Ti-site

```
TS–1-O-vacancy-near-Ti-site
   1.0000000000000000
    25.0000000000000000    0.0000000000000000    0.0000000000000000
     0.0000000000000000   25.0000000000000000    0.0000000000000000
     0.0000000000000000    0.0000000000000000   15.0000000000000000
   Si   H    Ti   O
     9    20     1    29
Selective dynamics
Direct
  0.3122148895594705  0.4344012695549822  0.5650504045441451   T   T   T
  0.5761627060686720  0.6722356353175130  0.4623697274946679   T   T   T
  0.6688163502014746  0.5915120317701508  0.4697846884253849   T   T   T
  0.6090308507775291  0.3687576721184342  0.5441055601248976   T   T   T
  0.3649693861471732  0.6556017430709673  0.4966132671251700   T   T   T
  0.4729331017749203  0.7026052910001539  0.5624034996627785   T   T   T
  0.3893999376398583  0.3672673177020656  0.4482998409760413   T   T   T
  0.6737394369821885  0.4790417408610779  0.5464372234209094   T   T   T
  0.3040720388588056  0.5491089939497442  0.4892627795445890   T   T   T
  0.3010500139999976  0.4317300029999984  0.7233800249999973   F   F   F
  0.6934200290000021  0.5972300339999990  0.3082500140000022   F   F   F
  0.4615900040000014  0.7978800200000009  0.5187299729999992   F   F   F
  0.5423300169999976  0.6596099850000030  0.3138700169999993   F   F   F
  0.2982299999999967  0.6987799839999980  0.5894700369999981   F   F   F
  0.6815799709999979  0.3271900180000031  0.4498699820000027   F   F   F
  0.3559799960000021  0.6921499629999985  0.3459599810000000   F   F   F
  0.6195799640000033  0.7602199549999966  0.4624100050000024   F   F   F
  0.5334699630000017  0.3036200140000034  0.3035600030000012   F   F   F
  0.4585900120000019  0.7208100129999977  0.7225099560000032   F   F   F
  0.7499300380000022  0.6203799820000029  0.5577800109999984   F   F   F
  0.3815000150000003  0.4080199810000025  0.2991900130000005   F   F   F
  0.2292499919999997  0.5780900190000011  0.5842699689999975   F   F   F
  0.7528199770000015  0.4432099910000034  0.4639799750000009   F   F   F
  0.2750900079999994  0.5506200029999988  0.3303200089999976   F   F   F
  0.6227800369999983  0.3279899979999996  0.6942800520000034   F   F   F
  0.7030100249999975  0.4885799790000007  0.6994600299999973   F   F   F
  0.4992300030000010  0.2347900010000004  0.5228199960000026   F   F   F
  0.2251000019999978  0.4023600009999981  0.5072600359999981   F   F   F
  0.3529399869999992  0.2768000029999982  0.4394400279999999   F   F   F
  0.5118464821332864  0.3339138687673540  0.4698626631980645   T   T   T
  0.3208599849999985  0.4413999940000011  0.6718000409999973   F   F   F
  0.5498200230000023  0.6469799799999976  0.3723199840000007   F   F   F
  0.6214679392005991  0.6320287665159867  0.5017030906384861   T   T   T
  0.6749800110000024  0.6003699870000005  0.3633599919999995   F   F   F
  0.6485199740000027  0.3336400219999973  0.4791400269999997   F   F   F
  0.3323699950000005  0.6960099790000029  0.5620100020000010   F   F   F
  0.3521200180000008  0.6668099979999980  0.3926699960000022   F   F   F
  0.6016399769999978  0.7300099950000032  0.4383399960000034   F   F   F
  0.3274642684184954  0.4905749580459613  0.5167098762834357   T   T   T
  0.6498667266614149  0.5305790803130463  0.4924544496180232   T   T   T
  0.7230200199999999  0.6026800160000008  0.5241199809999983   F   F   F
  0.5311065723815105  0.6770380956229545  0.5404220017572854   T   T   T
  0.6289216463837235  0.4319095667005683  0.5371038196851998   T   T   T
  0.3483639206374036  0.5938298019045760  0.5171362438000600   T   T   T
  0.4663999939999997  0.7025900269999994  0.6685000100000025   F   F   F
  0.3741100310000007  0.4009700009999975  0.3601799959999994   F   F   F
  0.4526937040072525  0.3759904379029351  0.4757094700762750   T   T   T
  0.5268199920000001  0.3263999939999991  0.3530499780000014   F   F   F
  0.2476900099999995  0.5575400159999973  0.5407900490000017   F   F   F
  0.7317699430000033  0.4651199719999966  0.5019999820000010   F   F   F
  0.2962900160000004  0.5516999820000024  0.3828599929999967   F   F   F
```

0.4278728590863210	0.6638591930933018	0.5179668270925930	T	T	T
0.4702600099999970	0.7609000400000028	0.5168300309999978	F	F	F
0.3529230051722934	0.3883389452805162	0.5301168207417675	T	T	T
0.6231600189999966	0.3545899960000014	0.6490999860000031	F	F	F
0.6822399899999994	0.4995299910000028	0.6496100110000000	F	F	F
0.2515999980000032	0.4157799910000008	0.5467299780000019	F	F	F
0.3796800229999988	0.3036400029999982	0.4347999889999983	F	F	F
0.4886499790000016	0.2711699870000004	0.5178099950000004	F	F	F

Structural parameter: TS–1 with O vacancy near Si-site

```
TS–1-O-vacancy-near-Ti-site
   1.0000000000000000
    25.0000000000000000    0.0000000000000000    0.0000000000000000
     0.0000000000000000   25.0000000000000000    0.0000000000000000
     0.0000000000000000    0.0000000000000000   15.0000000000000000
   Si   H    Ti   O
     9    20     1    29
Selective dynamics
Direct
  0.3116884881401134  0.4348084042020659  0.5653465209833115   T   T   T
  0.5695834659239125  0.6749468023958372  0.4667143441208239   T   T   T
  0.6669530622784166  0.5947330114383504  0.4703866813594217   T   T   T
  0.6171101321801160  0.3726253115125076  0.5475282145905354   T   T   T
  0.3663295830180076  0.6560441598558597  0.4970230602221619   T   T   T
  0.4781719208926987  0.7016382630035025  0.5598476251973210   T   T   T
  0.3863929957185590  0.3675734273334115  0.4486336174903940   T   T   T
  0.6754748615642470  0.4826171262365193  0.5456458439751017   T   T   T
  0.3043520629265046  0.5495044892607339  0.4893494507786329   T   T   T
  0.3010500139999976  0.4317300029999984  0.7233800249999973   F   F   F
  0.6934200290000021  0.5972300339999990  0.3082500140000022   F   F   F
  0.4615900040000014  0.7978800200000009  0.5187299729999992   F   F   F
  0.5423300169999976  0.6596099850000030  0.3138700169999993   F   F   F
  0.2982299999999967  0.6987799839999980  0.5894700369999981   F   F   F
  0.6815799709999979  0.3271900180000031  0.4498699820000027   F   F   F
  0.3559799960000021  0.6921499629999985  0.3459599810000000   F   F   F
  0.6195799640000033  0.7602199549999966  0.4624100050000024   F   F   F
  0.5334699630000017  0.3036200140000034  0.3035600030000012   F   F   F
  0.4585900120000019  0.7208100129999977  0.7225099560000032   F   F   F
  0.7499300380000022  0.6203799820000029  0.5577800109999984   F   F   F
  0.3815000150000003  0.4080199810000025  0.2991900130000005   F   F   F
  0.2292499919999997  0.5780900190000011  0.5842699689999975   F   F   F
  0.7528199770000015  0.4432099910000034  0.4639799750000009   F   F   F
  0.2750900079999994  0.5506200029999988  0.3303200089999976   F   F   F
  0.6227800369999983  0.3279899979999996  0.6942800520000034   F   F   F
  0.7030100249999975  0.4885799790000007  0.6994600299999973   F   F   F
  0.4992300030000010  0.2347900010000004  0.5228199960000026   F   F   F
  0.2251000019999978  0.4023600009999981  0.5072600359999981   F   F   F
  0.3529399869999992  0.2768000029999982  0.4394400279999999   F   F   F
  0.5039469306539957  0.3344057654113328  0.4660849577417352   T   T   T
  0.3208599849999985  0.4413999940000011  0.6718000409999973   F   F   F
  0.5498200230000023  0.6469799799999976  0.3723199840000007   F   F   F
  0.6172042110020399  0.6337648070394977  0.5038918844773406   T   T   T
  0.6749800110000024  0.6003699870000005  0.3633599919999995   F   F   F
  0.6485199740000027  0.3336400219999973  0.4791400269999997   F   F   F
  0.3323699950000005  0.6960099790000029  0.5620100020000010   F   F   F
  0.3521200180000008  0.6668099979999980  0.3926699960000022   F   F   F
  0.6016399769999978  0.7300099950000032  0.4383399960000034   F   F   F
  0.3276002118372929  0.4908632392083163  0.5170483770332766   T   T   T
  0.6494669650405740  0.5332296924844684  0.4933084089643600   T   T   T
  0.7230200199999999  0.6026800160000008  0.5241199809999983   F   F   F
  0.6332606346218057  0.4343626244817145  0.5362184242014294   T   T   T
  0.3488980421804158  0.5942216704142211  0.5170570684208826   T   T   T
  0.4663999939999997  0.7025900269999994  0.6685000100000025   F   F   F
  0.3741100310000007  0.4009700009999975  0.3601799959999994   F   F   F
  0.4476869693637298  0.3794221784212937  0.4761882621815872   T   T   T
  0.5268199920000001  0.3263999939999991  0.3530499780000014   F   F   F
  0.2476900099999995  0.5575400159999973  0.5407900490000017   F   F   F
  0.7317699430000033  0.4651199719999966  0.5019999820000010   F   F   F
  0.2962900160000004  0.5516999820000024  0.3828599929999967   F   F   F
  0.4302563148360363  0.6634399781563687  0.5178769779638728   T   T   T
```

0.4702600099999970	0.7609000400000028	0.5168300309999978	F	F	F
0.5541497751393152	0.3701418561754153	0.5263260620288932	T	T	T
0.3494146873360933	0.3868039710338067	0.5302770979539488	T	T	T
0.6231600189999966	0.3545899960000014	0.6490999860000031	F	F	F
0.6822399899999994	0.4995299910000028	0.6496100110000000	F	F	F
0.2515999980000032	0.4157799910000008	0.5467299780000019	F	F	F
0.3796800229999988	0.3036400029999982	0.4347999889999983	F	F	F
0.4886499790000016	0.2711699870000004	0.5178099950000004	F	F	F

References

1 Wilde, N., Worch, C., Suprun, W. & Gläser, R. Epoxidation of biodiesel with hydrogen peroxide over Ti-containing silicate catalysts. *Microporous and mesoporous materials* **164**, 182-189 (2012).

2 Grinberg, S., Kipnis, N., Linder, C., Kolot, V. & Heldman, E. Asymmetric bolaamphiphiles from vernonia oil designed for drug delivery. *European journal of lipid science and technology* **112**, 137-151 (2010).

3 Li, J., Ma, H., Sun, Q., Ying, W. & Fang, D. Effect of iron and phosphorus on HZSM-5 in catalytic cracking of 1-butene. *Fuel Processing Technology* **134**, 32-38 (2015).

4 Martins, N. C. *et al.* N-doped carbon quantum dots/TiO2 composite with improved photocatalytic activity. *Applied Catalysis B: Environmental* **193**, 67-74 (2016).
